# ARF6 Promotes AML Progression via Activation of PI3K/AKT/mTOR Signaling

**DOI:** 10.1002/cam4.70872

**Published:** 2025-04-24

**Authors:** Haitao Xu, Dangui Chen, Jia Lu, Long Zhong, Lihong Wang, Jian Ge

**Affiliations:** ^1^ Department of Hematology The First Affiliated Hospital of Anhui Medical University Hefei Anhui China; ^2^ Department of Hematology Anqing Municipal Hospital, Anqing Medical Center Affiliated to Anhui Medical University Anqing Anhui China

**Keywords:** AML, apoptosis, ARF6, cell cycle, PI3K/AKT/mTOR signaling, prognosis

## Abstract

**Background:**

Acute myeloid leukemia (AML) represents a highly aggressive hematological malignancy characterized by a poor prognosis and a pressing demand for innovative diagnostic and prognostic biomarkers. Recent studies have indicated that ADP‐ribosylation factor 6 (ARF6) is overexpressed across various cancer types; however, its specific role and implications in AML have yet to be thoroughly investigated.

**Methods:**

To elucidate the clinical relevance and functional mechanisms of ARF6 in AML, we conducted an integrated analysis utilizing RNA sequencing data from The Cancer Genome Atlas (TCGA) alongside clinical samples and AML cell lines. The diagnostic potential of ARF6 was assessed through receiver operating characteristic curve analysis, and logistic regression was employed to identify factors correlating with elevated ARF6 expression. Functional assays were performed to elucidate the effects of ARF6 modulation on apoptosis, cell cycle progression, and AML cell proliferation, while mechanistic investigations focused on the PI3K/AKT/mTOR signaling pathway, particularly in the context of pharmacological PI3K inhibition.

**Results:**

Our findings revealed a significant upregulation of ARF6 in AML compared to normal controls, with diagnostic efficacy indicated by an AUC of 0.793. Logistic regression analysis identified older age (> 60 years) and a higher white blood cell count (> 20 × 10^9^/L) as significant predictors of high ARF6 expression. Moreover, elevated ARF6 levels were independently associated with shorter overall survival (HR = 1.634, *p* = 0.045). Notably, ARF6 knockdown induced apoptosis and G0/G1 cell cycle arrest, whereas its overexpression yielded contrary effects. In addition, ARF6 activated the PI3K/AKT/mTOR pathway, which was abrogated by pharmacological PI3K inhibition.

**Conclusion:**

Collectively, our findings establish ARF6 as a valuable diagnostic and prognostic marker in AML, driving disease progression through the activation of the PI3K/AKT/mTOR pathway. These insights not only enhance our understanding of AML pathology but also underscore the potential of targeting ARF6 for therapeutic intervention in AML treatment paradigms. Future research should aim at evaluating the therapeutic implications of targeting ARF6 in combination with existing treatment modalities.

## 1 Introduction

Acute myeloid leukemia (AML) is a severe cancer marked by unregulated immature myeloid cell proliferation in the bone marrow and peripheral circulation [1, 2]. Notwithstanding progress in chemotherapy and stem cell transplantation, AML persists as one of the most prevalent forms of leukemia in adults, characterized by a dismal prognosis and a 5‐year overall survival rate of just 25%–40% [3, 4]. The AML pathophysiology contains a complex interaction of genetic and epigenetic modifications that impair normal hematopoietic cell growth and lead to leukemic transformation [5]. While several key molecular pathways, such as FLT3, RAS, and TP53 signaling, have been implicated in AML [6, 7, 8], there is an urgent need to clarify new processes that propel AML advancement in order to uncover new treatment targets and enhance patient outcomes.

ADP‐ribosylation factor 6 (ARF6) belongs to the ARF family of small GTPases, which are essential for controlling membrane trafficking, actin cytoskeleton remodeling, and cellular signaling [9, 10]. Mounting evidence has linked aberrant ARF6 activation to different perspectives of cancer advancement encompassing the capacity to migrate, invade, and metastasize [11, 12, 13]. Nevertheless, the potential ARF6 contribution to hematological malignancies like AML remains largely unexplored. A critical signaling cascade is the phosphatidylinositol 3‐kinase (PI3K)/AKT/mammalian target of rapamycin (mTOR) pathway that governs essential cellular activities, including growth, proliferation, metabolism, and survival [14, 15]. In human cancers, the PI3K/AKT/mTOR pathway is often aberrantly regulated, encompassing AML [16, 17]. Notwithstanding the advancement of several small molecule inhibitors aimed at the PI3K/AKT/mTOR pathway [18, 19], the upstream regulators and molecular mechanisms driving the aberrant PI3K/AKT/mTOR pathway in AML remain incompletely understood.

Intriguingly, recent studies have implicated ARF6 as a novel activator of PI3K and AKT in other cellular contexts [20, 21]. However, whether ARF6 can similarly modulate PI3K/AKT signaling in the context of AML and the potential functional consequences of such regulation have not been ascertained. Herein, we sought to address these key questions and test the hypothesis that ARF6 promotes AML progression via activating the PI3K/AKT/mTOR pathway. Our findings demonstrate that ARF6 is excessively overexpressed in AML and is linked to worse clinical outcomes. Mechanistically, we show that ARF6 forms a complex with PI3K and enhances its lipid kinase activity, leading to hyperactivation of downstream AKT/mTOR signaling. Importantly, pharmacological or genetic inhibition of PI3K/AKT/mTOR rescues the aggressive phenotypes induced by ARF6 overexpression, establishing this pathway as a critical mediator of ARF6's oncogenic effects in AML.

Collectively, our results uncover a novel ARF6‐PI3K/AKT/mTOR signaling axis that drives AML progression, suggesting that targeting this pathway may represent a promising therapeutic strategy for this challenging malignancy. This is the initial investigation linking the aberrant activation of ARF6 to the pathogenesis of AML. Our work significantly expands the understanding of ARF6's functions in cancer and lays the foundation for future investigations into the translational potential of ARF6 and its downstream effectors as biomarkers or therapeutic targets in AML.

## 2 Materials and Methods

### 2.1 Data Acquisition and Analysis

The Cancer Genome Atlas (TCGA) database (https://portal.gdc.cancer.gov/) was implemented to acquire the RNA sequencing data of AML patients, while the Gene Expression Omnibus (GEO) database (https://www.ncbi.nlm.nih.gov/geo/) was deployed to download the GSE9820 dataset. The raw data were investigated and normalized with the R packages “TCGAbiolinks.” [22] and “GEOquery” [23], respectively. ARF6 expression levels were compared between AML and normal samples using the R package “limma” [24]. Receiver Operating Characteristic (ROC) curve analysis was employed with the “pROC” R package [25] to ascertain the ARF6 diagnostic significance in AML. Kaplan–Meier analysis followed by log‐rank tests was utilized for prognostic analysis using the “survminer” package.

### 2.2 Clinical Samples

Bone marrow specimens were obtained from 5 AML patients at first diagnosis and 5 healthy controls at the Department of Hematology, Anqing Municipal Hospital. The clinical information of these patients and healthy controls was shown in Table 1. The Medical Ethics Committee of Anqing Municipal Hospital authorized the trial, and signed informed permission was acquired from all subjects. The diagnosis of AML was established based on the French‐American‐British (FAB) classification.

**TABLE 1:** The clinical information of AML patients and healthy controls.

Number	Gender	Age	FAB subtype	Karyotype	Molecular features
AML‐1	Male	54	M1	46, XY [20]	IDH2, NPM1
AML‐2	Male	46	M2	45, X, ‐Y, t (8; 21) (q22; q22) [10]	FLT3‐ITD, TET2
AML‐3	Male	48	M5	46, XY [20]	DNMT3A, NPM1
AML‐4	Female	60	M1	46, XX [20]	IDH1, PHF6, DNMT3A, NRAS, PTPN11
AML‐5	Female	55	M2	45, XX, −7 [20]	HOX11, EVI1, KRAS, PHF6, TET2
Control‐1	Male	46			
Control‐2	Male	40			
Control‐3	Male	52			
Control‐4	Female	53			
Control‐5	Female	58			

### 2.3 Cell Culture

The human AML cell lines HL‐60 and MV‐4‐11 were obtained from the Cell Bank of the Chinese Academy of Sciences in Shanghai, China. Cells were cultured in IMDM (Servicebio, G4640‐500ML) enriched with 20% fetal bovine serum (Excell Bio, FSP500), 100 U/mL of both ampicillin (Aladdin, A105484) and streptomycin sulfate (Aladdin, S105491) at 37°C in a humidified atmosphere containing 5% CO_2_.

### 2.4 Quantitative Real‐Time PCR (qRT‐PCR)

Total RNA was isolated from bone marrow specimens and AML cells with TRIzol reagent (Ambion, 15596‐026), aligning with the manufacturer's recommendations. The RNA purity and concentration were estimated with an ND‐100 spectrophotometer (Hangzhou Miu Instruments). cDNA was generated from 1.5 μg of total RNA with the HiScript II Q RT SuperMix (Vazyme, R223‐01). A QuantStudio 6 Real‐Time PCR System (ABI) was implemented to conduct qRT‐PCR with SYBR Green Master Mix (Vazyme, Q111‐02). The primers implemented for the amplification of human ARF6 were: forward, 5'‐GACGGTGACTTACAAAAATG‐3'; reverse, 5'‐CTGGATCTCGTGGGGTT‐3'. The PCR product size was 259 bp.

### 2.5 siRNA Transfection

2 mL of HL‐60 and MV‐4‐11 cells in the logarithmic phase were collected and seeded into a 6‐well plate at a concentration of 2.5 × 10^5^ cells/mL, respectively, and maintained at 37°C overnight. The cells were then transfected with 100 nM ARF6 siRNAs (si‐1‐584, si‐2‐803, si‐3‐895, si‐4‐1051) or negative control siRNA (si‐NC) using Lipofectamine 2000 (Invitrogen, 52887) that was diluted in opti‐MEM aligning with the manufacturer's guidelines. The siRNA sequences produced by GenePharma (Shanghai, China) are enumerated in Table S1. Cells were collected 24 h post‐transfection for future investigations. The knockdown effectiveness was ascertained using qRT‐PCR and Western blot (WB) experiments.

### 2.6 WB Experiment

The total protein was isolated with RIPA lysis buffer (Meilunbio, MA0151) that was supplemented with PMSF (Meilunbio, MB12707) and a phosphatase inhibitor (Meilunbio, MB12707). The BCA Protein Assay Kit (GBCBIO, G3422/G3522) was employed to quantify protein concentrations. SDS‐PAGE was employed to segregate similar protein volumes (40 μg), which were subsequently transferred to PVDF membranes (Millipore, IPVH00010). The membranes were incubated with primary antibodies that target ARF6 (1:2000, Sanying, 20225‐1‐AP), p‐mTOR, mTOR, p‐AKT, AKT (1:1000, Cell Signaling Technology), and GAPDH (1:1000, Affinity, AB‐P‐R 001) overnight at 4°C after a blocking step using 5% non‐fat milk. Subsequent to incubation with HRP‐conjugated secondary antibodies (1:10000, Boster Bio, BA1051/BA1054), the blots were quantified with an ECL kit (Affinity, KF8003) and then scanned with an SH‐523 chemiluminescence imaging system (Hangzhou Shenhua Technology).

### 2.7 Apoptosis Experiments

Annexin V‐APC/7‐AAD Apoptosis Detection Kit (KeyGEN BioTech, KGA1026, KGA1026) was employed to estimate cell apoptosis. Aligning with the manufacturer's recommendations, the cells were gathered 24 h following transfection, rinsed with PBS, and stained with Annexin V‐APC and 7‐AAD. The proportion of apoptotic cells was assessed with a CytoFLEX flow cytometer (FC) (Beckman Coulter).

### 2.8 Cell Cycle Analysis

The cell cycle distribution was ascertained with FC employing propidium iodide (PI) labeling. Transfected cells were fixed in pre‐chilled 80% ethanol at 4°C for a minimum of 4 h. Following PBS washing, cells were exposed to RNase (50 μg/mL) at 37°C for 30 min and then stained with PI (50 μg/mL, Sigma, P4170) at 4°C in the dark for 30 min. The DNA content was assessed with a CytoFLEX FC.

### 2.9 Cell Proliferation and Drug Treatment Assays

Cell proliferation was estimated via a Cell Counting Kit‐8 (CCK‐8) test (HYCEZMBIO, HYCCK8‐500 T). Twenty‐four hours post‐transfection, in 96‐well plates, cells were seeded at a density of 5 × 10^3^ cells per well. Typically, 10 μL of CCK‐8 solution was introduced to each well at 24, 48, 72, and 96 h. The Multiskan FC microplate reader (Thermo Scientific) was implemented to quantify the absorbance at 450 nm following 1–4 h of incubation at 37°C.

For drug treatment, cells were inoculated in 96‐well plates and subjected to different dosages of the PI3K inhibitor LY294002 (Selleck, USA) for 48 h. Cell viability was evaluated via the CCK‐8 test. ARF6 overexpression was achieved by transfecting cells with the pCMV3‐ARF6 plasmid (Sino Biological, China) using Lipofectamine 2000.

### 2.10 Plasmid Construction

The human ARF6 coding sequence was amplified from cDNA and cloned into the pLVX‐IRES‐ZsGreen1 lentiviral vector (Clontech) using the EcoRI and XbaI restriction sites. The primers implemented for cloning are presented in Table S2. The recombinant plasmid was verified by sequencing (Qingke Biotechnology).

### 2.11 Lentivirus Production and Transduction

293 T cells were co‐transfected with the pLVX‐IRES‐ZsGreen1‐ARF6 plasmid and packaging plasmids using Lipofectamine 2000. The supernatant containing lentiviral particles was gathered 48 h following transfection, filtered via a 0.45 μm filter, and concentrated with ultracentrifugation. HL‐60 and MV‐4‐11 cells were incubated with virus‐containing supernatant enriched with 8 μg/mL polybrene for 24 h to conduct lentiviral transduction. Cells that were stably transduced were selected using 2 μg/mL puromycin for a duration of 1 week.

### 2.12 Statistical Analysis

Statistical studies were conducted using R (version 4.2.1), SPSS 26.0, and GraphPad Prism 9.0 software. Data are stated as mean ± standard deviation (SD) derived from a minimum of three separate assays. Group variations were ascertained with Student's *t*‐test or one‐way ANOVA, accompanied by Tukey's post hoc test. The connections between ARF6 expression and clinicopathological features were determined with the chi‐square or Fisher's exact tests. Logistic regression was implemented to ascertain variables linked to elevated ARF6 expression.

Cox regression analyses, univariate and multivariate, were performed to ascertain independent predictive markers for overall survival. Survival curves were synthesized via the Kaplan–Meier technique and analyzed via the log‐rank test, with visualization achieved via the R package “survminer.” A nomogram including independent prognostic indicators was developed with the R package “rms,” and its efficacy was assessed by calibration plots. A *p* value of below 0.05 was deemed significant.

## 3 Results

### 3.1 ARF6 Was Elevated in Multiple Cancers, Particularly in AML

**FIGURE 1:**
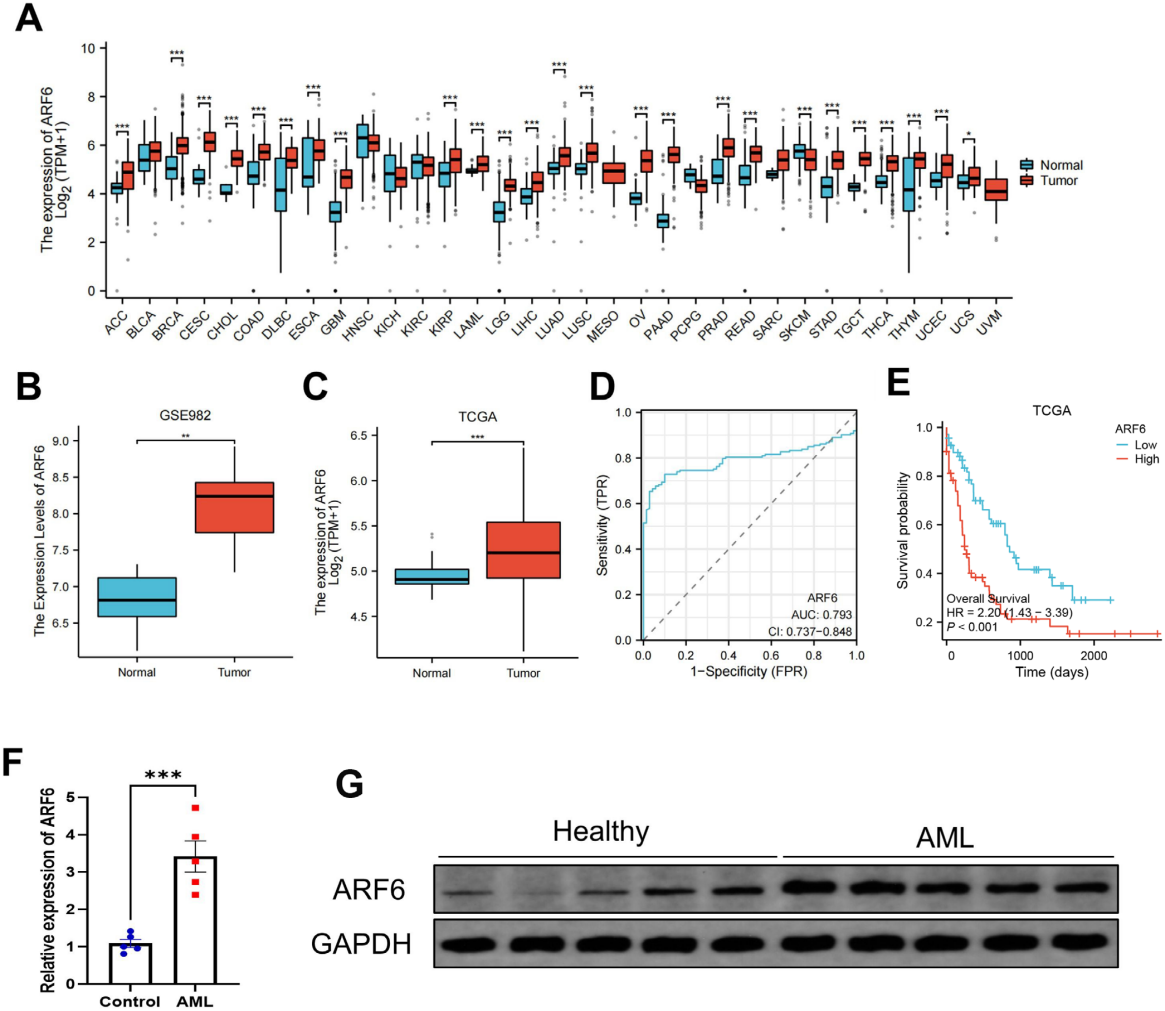
ARF6 expression in various cancer types and its diagnostic value in AML. (A) The expression of ARF6 across different cancer types based on the TCGA dataset. The box plots show log2(TPM + 1) expression levels in tumor tissues (red) contrasted with normal tissues (blue). TCGA‐LAML refers to a cohort of patients with AML. (B–C) Expression levels of ARF6 in AML patients versus healthy controls from the GSE982 and TCGA datasets. (D) ROC curve analysis of ARF6 expression to differentiate AML patients from healthy controls. The AUC value was 0.793, indicating a good diagnostic performance. (E) Kaplan–Meier survival plot of ARF6 expression based on TCGA dataset. (F) Relative expression of ARF6 in AML patients contrasted with healthy controls. Data are stated as mean ± SD. (G) WB experiment of ARF6 protein levels in healthy controls and AML patients, with GAPDH as a loading control. *p < 0.05, **p < 0.01, ***p < 0.001.

Analysis of TCGA data reported that ARF6 expression is significantly elevated in several tumor types, including AML (Figure 1A). This upregulation was further confirmed in AML patients compared to healthy controls using the GSE982 dataset (Figure 1B) and the TCGA dataset (Figure 1C). Further ROC curve analysis demonstrated that ARF6 has good diagnostic potential (Figure 1D). Kaplan–Meier survival plot of the TCGA dataset showed that ARF6 high expression was associated with poor prognosis of AML patients (Figure 1E). Additional qRT‐PCR and WB experiments validated the higher ARF6 mRNA and protein levels, respectively, in AML samples contrasted with controls (*p* < 0.001) (Figure 1F,G).

### 3.2 siRNA‐Mediated Knockdown of ARF6 in AML Cell Lines HL‐60 and MV‐4‐11

**FIGURE 2:**
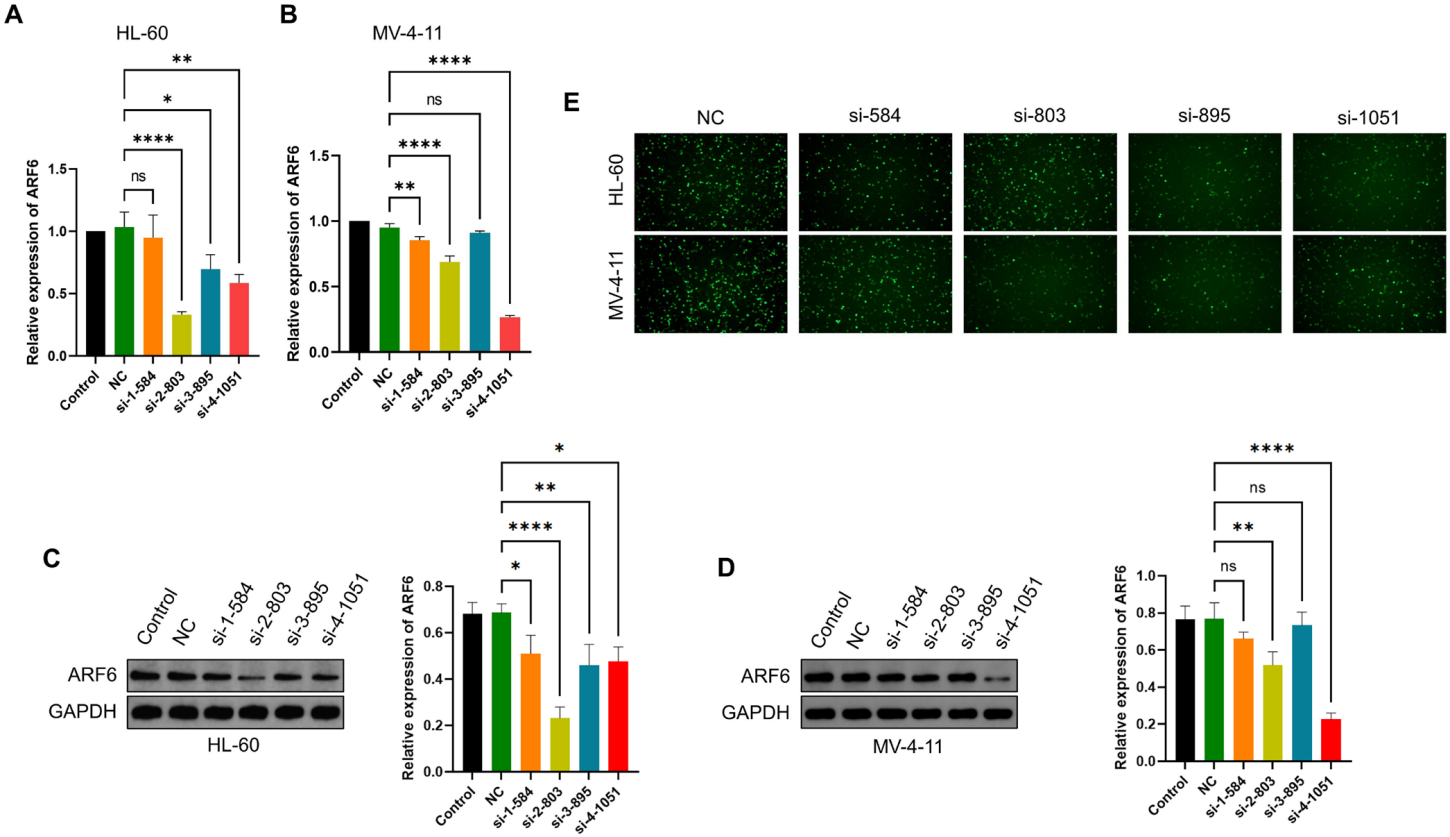
ARF6 knockdown in AML cell lines. (A) Relative expression of ARF6 in HL‐60 cells after transfection with different siRNAs targeting ARF6 (si‐1‐584, ‐2‐803, ‐3‐895, ‐4‐1051) compared to control and NC (non‐targeting control). (B) Relative expression of ARF6 in MV‐4‐11 cells after transfection with the same set of siRNAs. (C) WB experiment of ARF6 protein levels in HL‐60 cells transfected with different siRNAs compared to control and NC, with GAPDH as a loading control. (D) WB experiment of ARF6 protein levels in MV‐4‐11 cells transfected with different siRNAs contrasted with control and NC. (E) Fluorescence microscopy images showing the efficiency of siRNA‐mediated knockdown of ARF6 in HL‐60 and MV‐4‐11 cells. Cells were stained with a fluorescent dye to visualize ARF6 expression. *p < 0.05, **p < 0.01, ****p < 0.0001, ns: Not significant.

To investigate the effects of ARF6 knockdown, HL‐60 and MV‐4‐11 AML cell lines were transfected with four various siRNAs targeting ARF6 (si‐1‐584, ‐2‐803, ‐3‐895, ‐4‐1051). qRT‐PCR, WB, and fluorescence microscopy analyses confirmed that si‐2‐803, si‐895, and si‐4‐1051 showed the most substantial knockdown efficiency in HL‐60 cells, while si‐2‐803 and si‐4‐1051 exhibited the highest knockdown efficiency in MV‐4‐11 cells (*p* < 0.0001) (Figure 2A–E). Thus, si‐2‐803 and si‐4‐1051 were selected for subsequent experiments.

### 3.3 ARF6 Knockdown Induces Apoptosis and Alters Cell Cycle Advancement in AML Cell Lines

**FIGURE 3:**
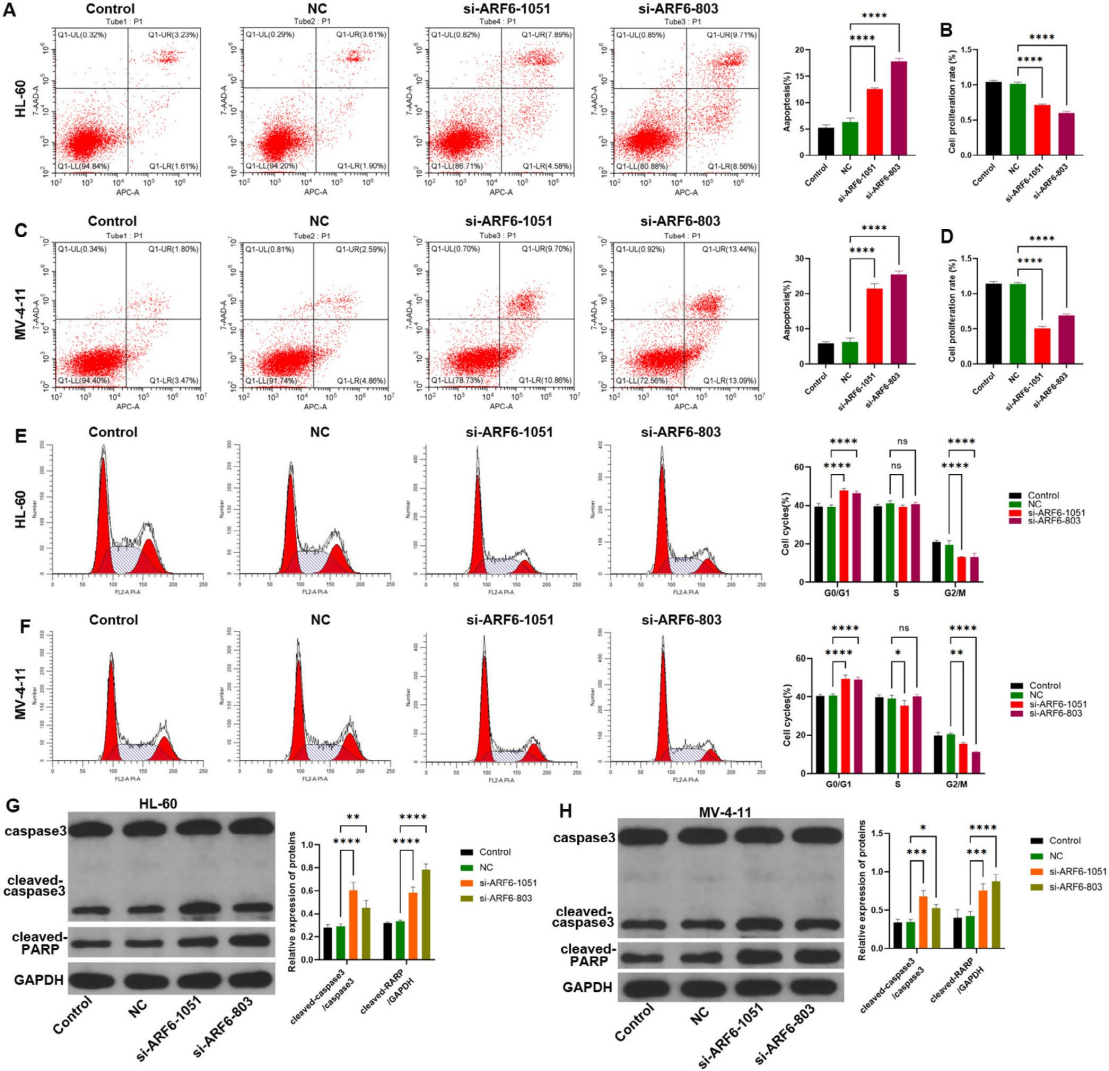
Implications of ARF6 knockdown on apoptosis and cell cycle in AML cell lines. (A) FC experiment to investigate apoptosis in HL‐60 cells that have been transfected with control, NC (non‐targeting control), si‐ARF6‐1051, or si‐ARF6‐803. The bar graph illustrates the proportion of apoptotic cells. (B) Cell proliferation rate of HL‐60 cells after ARF6 knockdown compared to control and NC. (C) FC analysis of apoptosis in MV‐4‐11 cells transfected with control, NC, si‐ARF6‐1051, or si‐ARF6‐803. The bar graph illustrates the proportion of apoptotic cells. (D) Cell proliferation rate of MV‐4‐11 cells after ARF6 knockdown compared to control and NC. (E, F) Cell cycle distribution analysis using FC in HL‐60 and MV‐4‐11 cells that have been transfected with control, NC, si‐ARF6‐1051, or si‐ARF6‐803. (G, H) Western blot analysis of apoptosis‐related proteins expression in HL‐60 and MV‐4‐11 cells that have been transfected with control, NC, si‐ARF6‐1051, or si‐ARF6‐803. A bar graph is presented to illustrate the percentage of cells in the G0/G1, S, and G2/M phases. *p < 0.05, **p < 0.01, ***p < 0.001, ****p < 0.0001, ns: Not significant.

FC experiment revealed that ARF6 knockdown by si‐803 and si‐1051 significantly increased apoptosis in both HL‐60 and MV‐4‐11 cells (Figure 3A,C). Both si‐ARF6‐1051 and si‐ARF6‐803 significantly mitigated cell proliferation rates in both cell lines (*p* < 0.0001) (Figure 3B,D). Cell cycle analysis reported that knocking down ARF6 significantly elevated the G0/G1 phase and a corresponding hindrance in the G2/M phase in both HL‐60 and MV‐4‐11 cells (*p* < 0.0001), while the S phase remained unchanged (Figure 3E,F). In addition, WB assay showed that both si‐ARF6‐1051 and si‐ARF6‐803 promoted the cleavage of caspase3 and RARP (Figure 3G,H).

### 3.4 Effect of ARF6 Overexpression and PI3K Inhibition on Cell Proliferation in AML Cell Lines

**FIGURE 4:**
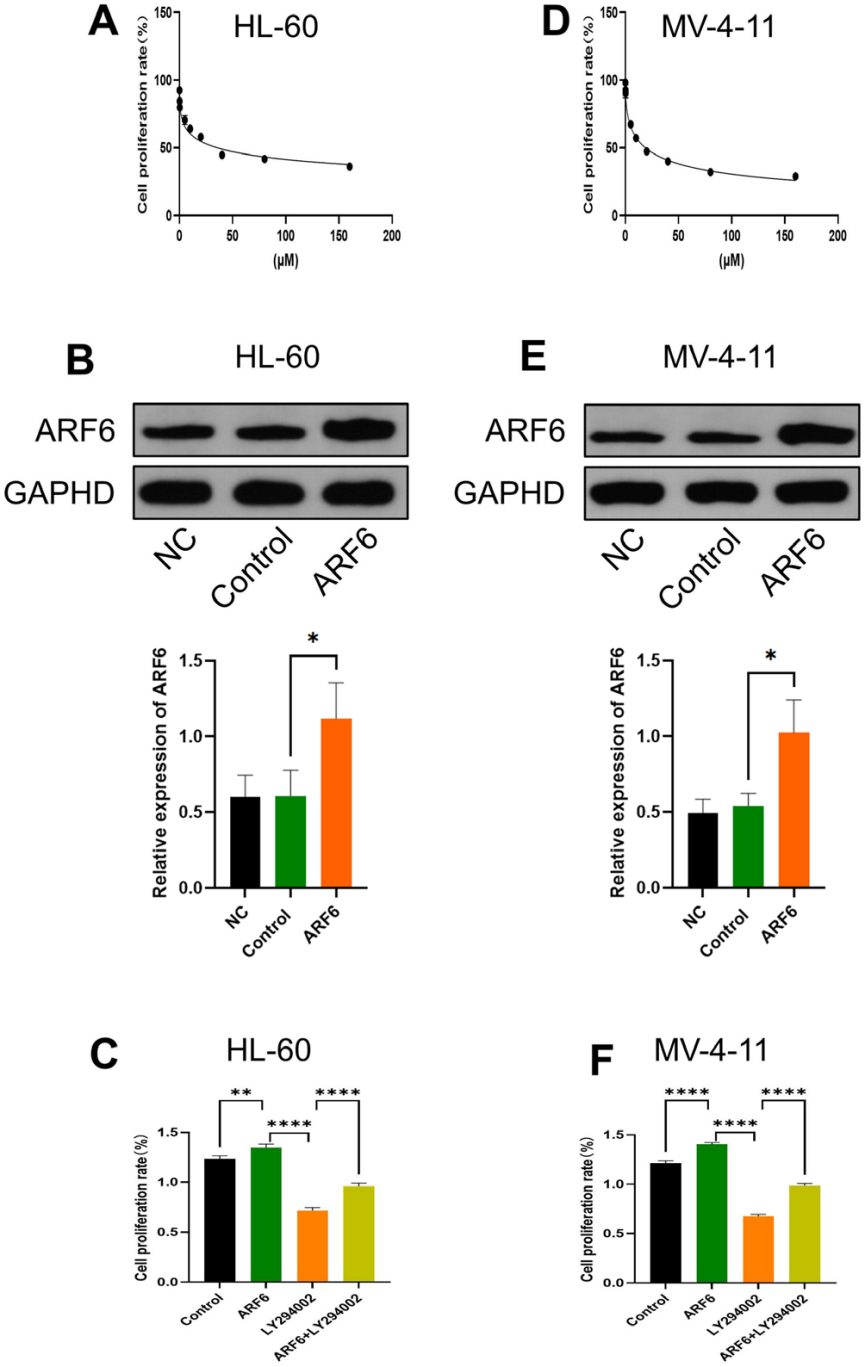
Impact of ARF6 overexpression and PI3K inhibitor LY294002 on cell proliferation in AML cell lines. (A) Dose–response curve of HL‐60 cells treated with various LY294002 concentration. Cell proliferation rate is stated as a proportion of the control. (B) WB experiment of ARF6 protein levels in HL‐60 cells under different conditions: NC (non‐targeting control), control, and ARF6 overexpression, with GAPDH as a loading control. (C) Cell proliferation rates of HL‐60 cells under different conditions: Control, ARF6 overexpression, LY294002 treatment, and ARF6 overexpression combined with LY294002 treatment. (D) Dose–response curve of MV‐4‐11 cells treated with different concentrations of LY294002. Cell proliferation rate is stated as a proportion of the control. (E) WB study of ARF6 protein levels in MV‐4‐11 cells under different conditions: NC, control, and ARF6 overexpression, with GAPDH as a loading control. (F) Cell proliferation rates of MV‐4‐11 cells under different conditions: Control, ARF6 overexpression, LY294002 treatment, and ARF6 overexpression combined with LY294002 treatment. (B, C, E, F) Data are presented as mean ± SD. *p < 0.05, **p < 0.01, ****p < 0.0001.

Furthermore, we investigated the effect of ARF6 overexpression in AML cells and the impact of PI3K inhibition on ARF6 overexpression‐mediated biological function. Dose–response curves showed that the PI3K inhibitor LY294002 hindered cell proliferation in a dose‐dependent way in HL‐60 and MV‐4‐11 cells (Figure 4A,D). The IC50 of HL‐60 cells (37 μM) and MV‐4‐11 cells (18 μM) was chosen for subsequent experiments. The expression of ARF6 was then upregulated in HL‐60 and MV‐4‐11 cell lines (Figure 4B,E). CCK‐8 assay showed that ARF6 overexpression alone significantly increased cell proliferation in cell lines (*p* < 0.01), while LY294002 significantly reduced proliferation compared to ARF6 overexpression. The combination of ARF6 overexpression and LY294002 treatment significantly increased proliferation compared to LY294002 treatment alone (*p* < 0.0001) (Figure 4C,F).

### 3.5 Combined Effects of ARF6 Overexpression and PI3K Inhibition on Apoptosis and Cell Cycle Progression

**FIGURE 5:**
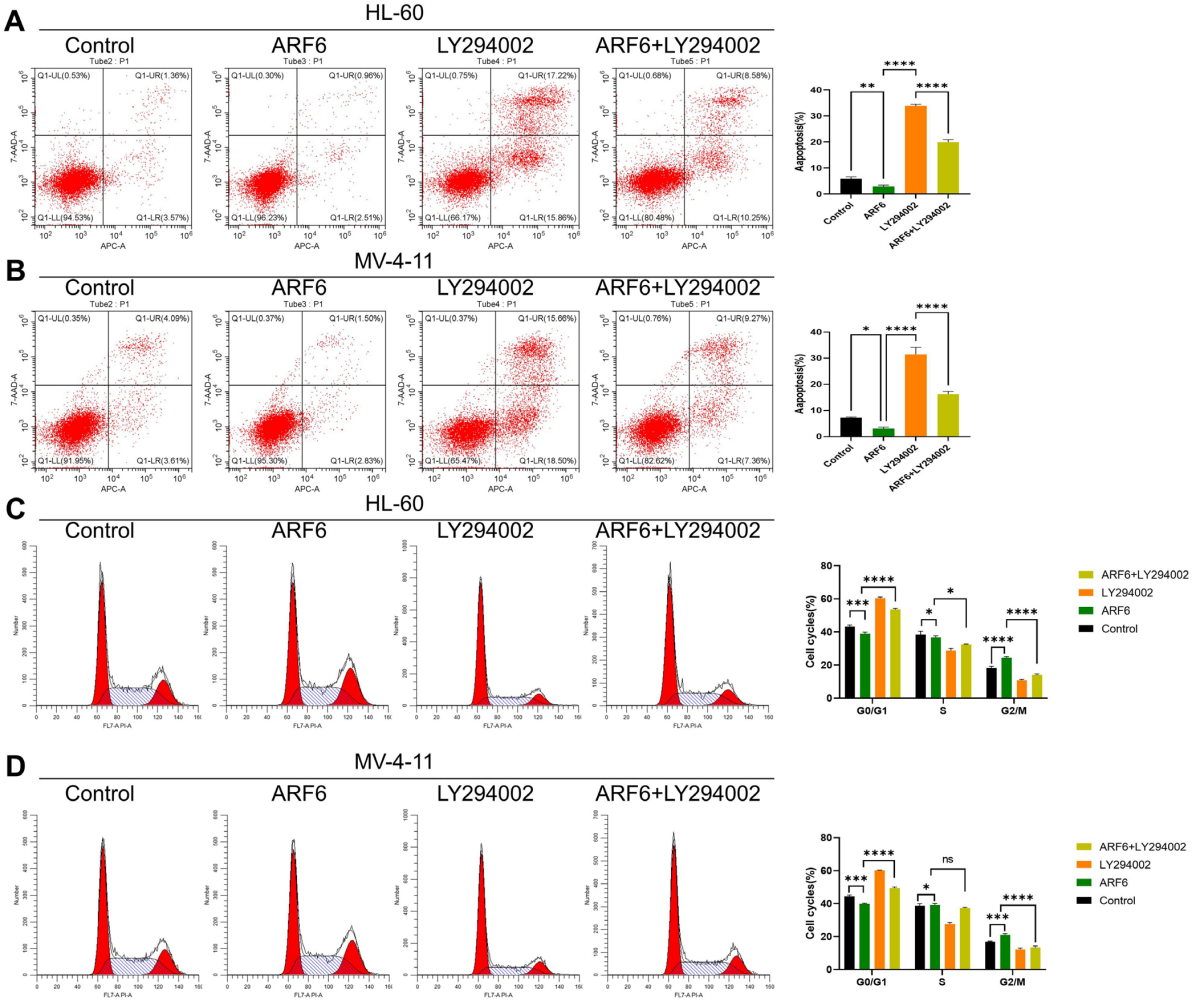
Combined implications of ARF6 overexpression and PI3K inhibitor LY294002 on apoptosis and cell cycle in AML cell lines. (A) FC experiment of apoptosis in HL‐60 cells under different conditions: Control, ARF6 overexpression, LY294002 treatment, and ARF6 overexpression combined with LY294002 treatment. The bar graph illustrates the proportion of apoptotic cells. (B) FC analysis of apoptosis in MV‐4‐11 cells under the same conditions as in (A). The bar graph illustrates the proportion of apoptotic cells. (C, D) Cell cycle distribution in HL‐60 and MV‐4‐11 cells under various conditions: Control, ARF6 overexpression, LY294002 treatment, and ARF6 overexpression combined with LY294002 treatment, examined with FC. A bar graph is presented to illustrate the percentage of cells in the G0/G1, S, and G2/M phases. *p < 0.05, **p < 0.01, ***p < 0.001, ****p < 0.0001, ns: Not significant.

FC analysis showed that in both HL‐60 and MV‐4‐11 cells, ARF6 overexpression resulted in a decrease in apoptosis in AML cells, which has been reversed by LY294002 (Figure 5A,B). Cell cycle analysis revealed that ARF6 overexpression alone declined the G0/G1 phase and elevated the S phase, while LY294002 treatment induced G0/G1 arrest. The combination treatment enhanced G0/G1 arrest and reduced the G2/M phase in cell lines (*p* < 0.0001) (Figure 5C,D).

### 3.6 ARF6 Overexpression and PI3K Inhibition Affect mTOR and AKT Signaling Pathways in AML Cell Lines

**FIGURE 6:**
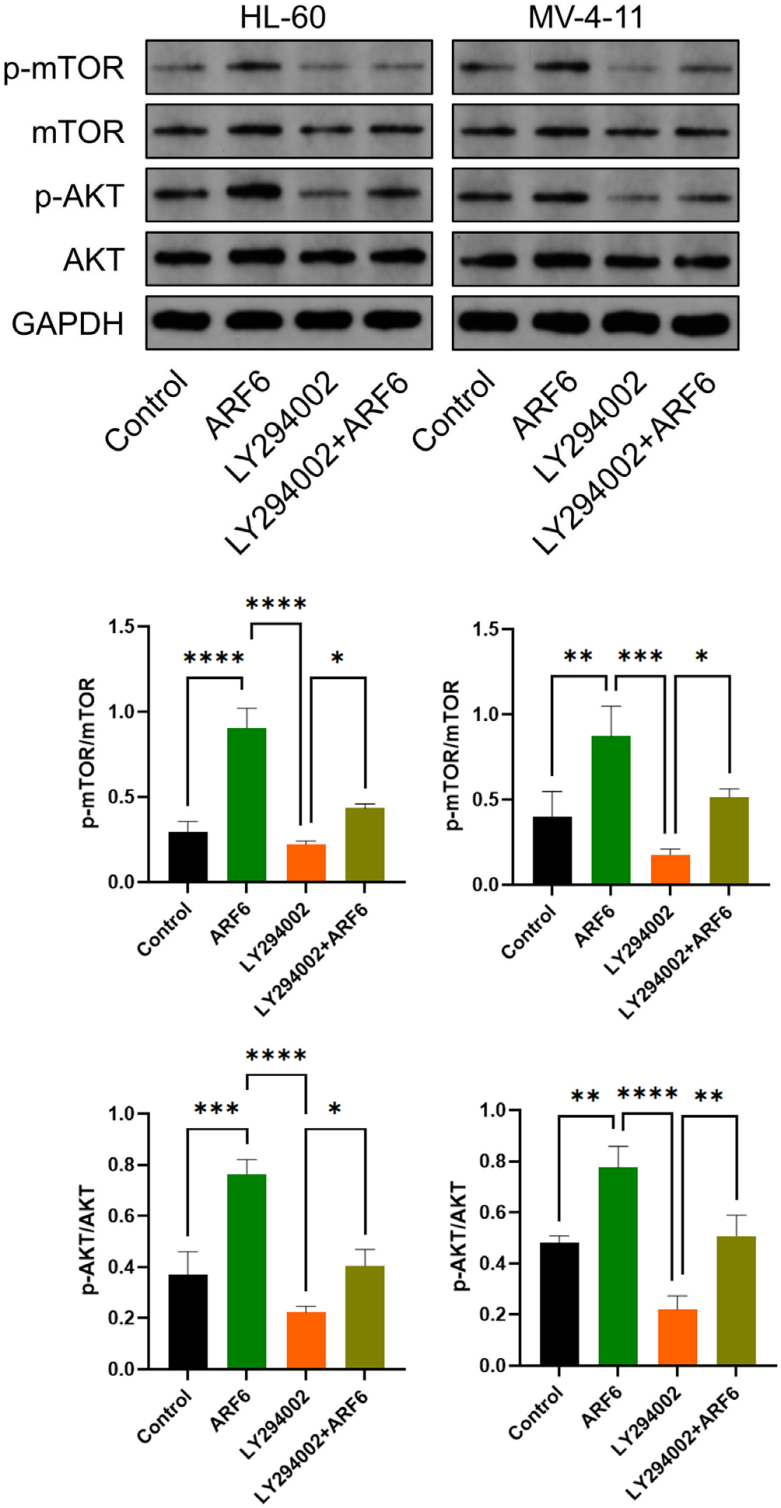
Effect of ARF6 overexpression and PI3K inhibitor LY294002 on mTOR and AKT signaling pathways in AML cell lines. WB analysis of phosphorylated mTOR (p‐mTOR), total mTOR, phosphorylated AKT (p‐AKT), total AKT, and GAPDH in HL‐60 and MV‐4‐11 cells under different conditions: Control, ARF6 overexpression, LY294002 treatment, and ARF6 overexpression combined with LY294002 treatment. GAPDH operated as a loading control. *p < 0.05, **p < 0.01, ***p < 0.001, ****p < 0.0001.

WB analysis revealed that ARF6 overexpression led to increased phosphorylated mTOR (p‐mTOR) and phosphorylated AKT (p‐AKT) levels in HL‐60 and MV‐4‐11 cells, indicating activation of the mTOR and AKT signaling pathways compared to the control (Figure 6). Treatment with the PI3K inhibitor LY294002 effectively mitigated the p‐mTOR and p‐AKT levels, demonstrating its inhibitory effect on these pathways. Notably, when ARF6 overexpression was combined with LY294002 treatment, the reduction in p‐mTOR and p‐AKT levels was still observed, suggesting that LY294002 can counteract the mTOR and AKT pathways activation induced by ARF6 overexpression. Total levels of mTOR and AKT remained relatively unchanged across all conditions.

### 3.7 Clinical and Genetic Characteristics Associated With ARF6 Expression in AML

We also analyze the clinical and genetic characteristics associated with ARF6 expression in AML using the TCGA dataset. Patients were divided into low and high expression of ARF6 groups according to the median expression of ARF6. As shown in Table 2, high ARF6 expression was significantly linked to older age (> 60 years, *p* = 0.002) and greater white blood cell (WBC) count (> 20 × 10^9^/L, *p* = 0.017). ARF6 expression did not exhibit a significant connection with gender, race, bone marrow blasts, peripheral blood blasts, cytogenetic risk, specific cytogenetic abnormalities, or mutations in FLT3, IDH1, RAS, or NPM1.

**TABLE 2:** Correlation between ARF6 expression and clinicopathological characteristics in LAML samples derived from the TCGA database.

Characteristics	Low expression of ARF6	High expression of ARF6	*p*
*n*	75	75	
Gender, *n* (%)			0.622
Female	35 (23.3%)	32 (21.3%)	
Male	40 (26.7%)	43 (28.7%)	
Race, *n* (%)			0.557
Asian and Black or African American	8 (5.4%)	6 (4%)	
White	66 (44.3%)	69 (46.3%)	
Age, *n* (%)			0.002
≤ 60	53 (35.3%)	34 (22.7%)	
> 60	22 (14.7%)	41 (27.3%)	
WBC count (×10^9^/L), *n* (%)			0.017
≤ 20	45 (30.2%)	31 (20.8%)	
> 20	29 (19.5%)	44 (29.5%)	
BM blasts (%), *n* (%)			0.242
≤ 20	26 (17.3%)	33 (22%)	
> 20	49 (32.7%)	42 (28%)	
PB blasts (%), *n* (%)			0.141
≤ 70	40 (26.7%)	31 (20.7%)	
> 70	35 (23.3%)	44 (29.3%)	
Cytogenetic risk, *n* (%)			0.505
Favorable	18 (12.2%)	12 (8.1%)	
Intermediate/normal	39 (26.4%)	43 (29.1%)	
Poor	18 (12.2%)	18 (12.2%)	
Cytogenetics, *n* (%)			0.672
Normal	34 (28.8%)	35 (29.7%)	
inv (16) and t (8; 21)	8 (6.8%)	7 (5.9%)	
t (15; 17)	7 (5.9%)	3 (2.5%)	
Complex	13 (11%)	11 (9.3%)	
FLT3 mutation, *n* (%)			0.133
Negative	54 (37%)	47 (32.2%)	
Positive	18 (12.3%)	27 (18.5%)	
IDH1 R132 mutation, *n* (%)			0.384
Negative	66 (44.6%)	69 (46.6%)	
Positive	8 (5.4%)	5 (3.4%)	
IDH1 R140 mutation, *n* (%)			0.228
Negative	70 (47.3%)	66 (44.6%)	
Positive	4 (2.7%)	8 (5.4%)	
IDH1 R172 mutation, *n* (%)			0.477
Negative	72 (48.6%)	74 (50%)	
Positive	2 (1.4%)	0 (0%)	
RAS mutation, *n* (%)			0.731
Negative	71 (47.7%)	70 (47%)	
Positive	3 (2%)	5 (3.4%)	
NPM1 mutation, *n* (%)			0.181
Negative	61 (40.9%)	55 (36.9%)	
Positive	13 (8.7%)	20 (13.4%)	

### 3.8 Factors Associated With High ARF6 Expression in AML Patients

Logistic regression analysis identified age greater than 60 years (OR = 2.905, 95% CI: 1.481–5.698, *p* = 0.002) and elevated WBC count (> 20 × 10^9^/L) (OR = 2.202, 95% CI: 1.144–4.240, *p* = 0.018) as factors significantly associated with high ARF6 expression in AML patients. Other clinical and genetic factors showed no significant associations (Table 3).

**TABLE 3:** Logistic analysis for determining the relationship between clinicopathological factors of LAML and ARF6 expression.

Characteristics	Total (*N*)	OR (95% CI)	*p*
Gender (male vs. female)	150	1.176 (0.617–2.240)	0.622
Race (White vs. Asian and Black or African American)	149	1.394 (0.459–4.234)	0.558
Age (> 60 vs. ≤ 60)	150	2.905 (1.481–5.698)	**0.002**
WBC count (×10^9^/L) (> 20 vs. ≤ 20)	149	2.202 (1.144–4.240)	**0.018**
BM blasts (%) (> 20 vs. ≤ 20)	150	0.675 (0.349–1.305)	0.243
PB blasts (%) (> 70 vs. ≤ 70)	150	1.622 (0.850–3.094)	0.142
Cytogenetic risk (poor and intermediate/normal vs. favorable)	148	1.605 (0.711–3.626)	0.255
FLT3 mutation (positive vs. negative)	146	1.723 (0.845–3.516)	0.135
RAS mutation (positive vs. negative)	149	1.690 (0.389–7.344)	0.484
NPM1 mutation (positive vs. negative)	149	1.706 (0.776–3.751)	0.184
IDH1 R132 mutation (positive vs. negative)	148	0.598 (0.186–1.921)	0.388
IDH1 R140 mutation (positive vs. negative)	148	2.121 (0.610–7.377)	0.237
IDH1 R172 mutation (positive vs. negative)	148	1.343 (0.432–4.569)	0.306

*Note:* Bold values states that *p* < 0.05 was considered statistically significant.

### 3.9 Prognostic Significance of ARF6 Expression and Other Clinical Factors in AML Patients

Univariate and multivariate Cox regression studies verified that high ARF6 expression was independently linked to raised death hazard in AML patients (multivariate HR = 1.634, 95% CI: 0.990–2.695, *p* = 0.045). Age over 60 years and poor cytogenetic risk were also significant independent prognostic factors for overall survival. Gender, race, WBC count, FLT3 mutation, and mutations in IDH1, RAS, and NPM1, as well as bone marrow and peripheral blood blasts, did not show significant associations with overall survival (Table 4).

**TABLE 4:** Univariate and multivariate analysis of the correlation between ARF6 expression and overall survival in patients with AML.

Characteristics	Total (*N*)	Univariate analysis		Multivariate analysis	
		Hazard ratio (95% CI)	*p*	Hazard ratio (95% CI)	*p*
Gender	139				
Female	62	Reference			
Male	77	1.024 (0.671–1.564)	0.912		
Race	138				
Asian and Black or African American	11	Reference			
White	127	1.200 (0.485–2.966)	0.693		
Age	139				
≤ 60	78	Reference		Reference	
> 60	61	3.321 (2.156–5.116)	**< 0.001**	2.793 (1.676–4.653)	**< 0.001**
WBC count (×10^9^/L)	138				
≤ 20	74	Reference			
> 20	64	1.156 (0.757–1.764)	0.503		
BM blasts (%)	139				
≤ 20	58	Reference			
> 20	81	1.159 (0.754–1.780)	0.502		
PB blasts (%)	139				
≤ 70	65	Reference			
> 70	74	1.224 (0.802–1.869)	0.349		
Cytogenetic risk	137				
Favorable	30	Reference		Reference	
Intermediate/normal	76	2.934 (1.487–5.791)	**0.002**	2.358 (0.998–5.237)	**0.008**
Poor	31	4.124 (1.927–8.823)	**< 0.001**	3.674 (1.416–7.684)	**< 0.001**
FLT3 mutation	135				
Negative	96	Reference			
Positive	39	1.266 (0.798–2.009)	0.316		
IDH1 R132 mutation	137				
Negative	125	Reference			
Positive	12	0.586 (0.237–1.448)	0.247		
IDH1 R140 mutation	137				
Negative	126	Reference			
Positive	11	1.129 (0.564–2.258)	0.733		
RAS mutation	138				
Negative	130	Reference			
Positive	8	0.642 (0.235–1.756)	0.388		
NPM1 mutation	138				
Negative	105	Reference			
Positive	33	1.134 (0.704–1.826)	0.606		
ARF6	139				
Low	69	Reference		Reference	
High	70	2.202 (1.430–3.391)	**< 0.001**	1.634 (0.990–2.695)	**0.045**

*Note:* Bold values states that *p* < 0.05 was considered statistically significant.

### 3.10 Creation and Verification of a Nomogram for Forecasting Overall Survival in AML Patients

**FIGURE 7:**
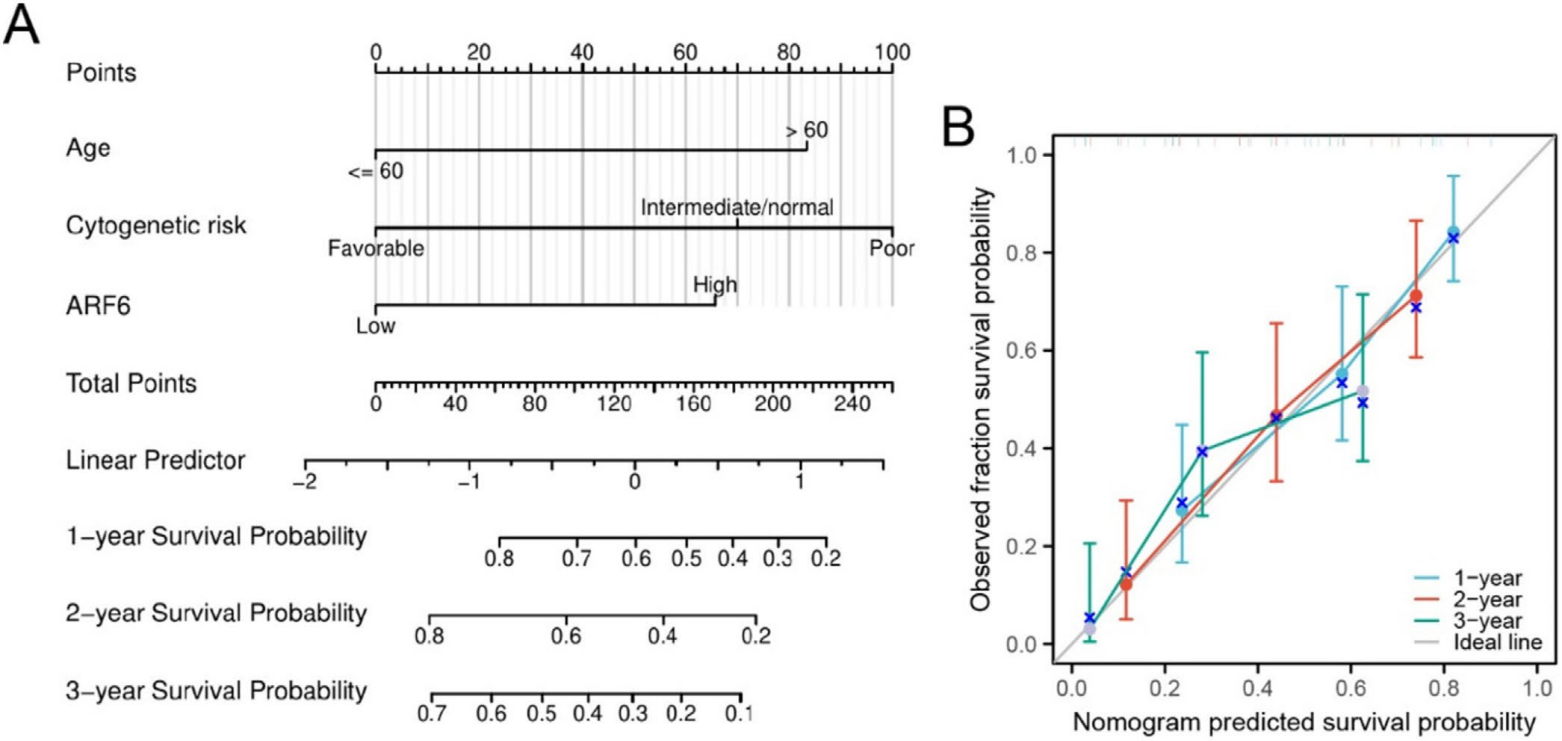
Nomogram for forecasting overall survival in AML patients. (A) Nomogram for anticipating 1‐, 2‐, and 3‐year overall survival probabilities in AML patients. The nomogram integrates age, cytogenetic risk, and ARF6 expression levels. Points are assigned for each variable, and the total points correspond to predicted survival probabilities. (B) Calibration plot shows the nomogram for forecasting overall survival at 1‐, 2‐, and 3‐year intervals. The *x*‐axis denotes the survival probability anticipated by the nomogram, whereas the *y*‐axis indicates the actual survival probability. The ideal line signifies an optimal predictive model, whereas the colorful lines illustrate the performance of the nomogram.

A nomogram integrating age, cytogenetic risk, and ARF6 expression levels was created to anticipate 1‐, 2‐, and 3‐year overall survival probabilities in AML patients (Figure 7A). A calibration plot confirmed that the nomogram predictions closely aligned with the observed survival probabilities, particularly for the 1‐, 2‐, and 3‐year time points (Figure 7B).

## 4 Discussion

ARF6, a relatively obscure member of the ADP‐ribosylation factor (ARF) family of small GTPases, has lately gained attention as a significant component in cancer biology. While its siblings, such as ARF1 [26] and ARF5 [27], have been widely investigated for their contributions to intracellular trafficking and organelle function, ARF6 has quietly been making waves in the field of cancer research [28, 29]. Nevertheless, the ARF6 contribution to AML is still largely unexplored. Herein, we examined the ARF6 clinical value and functional mechanisms in AML. Our findings reveal that ARF6 is elevated in AML patients and links to a worse prognosis. Moreover, we demonstrate that ARF6 enhances the AML cell's proliferation and survival while suppressing apoptosis via activating the PI3K/AKT/mTOR pathway.

The predictive value of ARF6 expression in AML is a key finding of our study. Univariate and multivariate Cox regression studies reported that high ARF6 expression independently predicted shorter overall survival (HR = 1.634, *p* = 0.045), even after adjusting for established prognostic factors such as age and cytogenetic risk. These outcomes are aligned with prior investigations reporting the prognostic value of ARF6 in other malignancies, such as renal cancer and hepatocellular carcinoma [29, 30]. To enhance the clinical utility of ARF6, we developed a nomogram integrating its expression with age and cytogenetic risk, which accurately predicted patient survival probabilities. The nomogram demonstrated good calibration and could potentially guide personalized treatment decisions and follow‐up strategies in clinical practice.

Our functional experiments provide mechanistic insights into the oncogenic role of ARF6 in AML. siRNA‐mediated knockdown of ARF6 stimulated apoptosis, G0/G1 cell cycle arrest, and hindered AML cell line proliferation, while ARF6 overexpression exhibited opposing effects. These findings are in line with the pro‐survival and proliferative contributions of ARF6 reported in other cancers [31, 32]. The ability of ARF6 to promote AML cell survival and proliferation suggests its potential involvement in chemoresistance and disease relapse [33, 34], which are major challenges in AML management.

Mechanistically, we found that ARF6 exerts its oncogenic effects in AML via the PI3K/AKT/mTOR pathway activation. ARF6 overexpression elevated the phosphorylated AKT and mTOR levels, indicating pathway activation, while pharmacological inhibition of PI3K abrogated these effects. The PI3K/AKT/mTOR pathway is often aberrantly regulated in AML and is involved in leukemogenesis, cell survival, and chemoresistance [35, 36]. Our findings provide a novel link between ARF6 and PI3K/AKT/mTOR signaling in AML, revealing that ARF6 may operate as a possible therapeutic target for pathway inhibition. Small molecule inhibitors targeting ARF6 or its downstream effectors could be explored as a promising strategy for AML treatment.

Our investigation possesses many limitations that warrant consideration. The sample size of our clinical cohort was somewhat limited, and larger multicenter studies are required to validate the predictive significance of ARF6 in AML. Second, our functional experiments were limited to in vitro assays using AML cell lines. Future studies should employ in vivo models to investigate the consequence of ARF6 manipulation on leukemia initiation, progression, and response to therapy. Third, while we focused on the PI3K/AKT/mTOR pathway, ARF6 may interact with other signaling cascades in AML, which remain to be explored.

Based on our findings, several future research directions can be proposed. First, the upstream regulators and downstream effectors of ARF6 in AML need to be identified to gain a more comprehensive understanding of its signaling network. Second, the therapeutic potential of targeting ARF6 should be investigated using both genetic and pharmacological approaches in preclinical AML models. Combination strategies with existing chemotherapeutic agents or targeted therapies could also be explored. Third, the role of ARF6 in other hematological malignancies merits investigation, given its involvement in multiple solid tumors [37, 38].

In conclusion, our study identifies ARF6 as a new diagnostic and predictive biomarker in AML that promotes disease advancement through the PI3K/AKT/mTOR pathway activation. The developed nomogram integrating ARF6 expression with clinical factors accurately predicts patient survival and may guide risk stratification and treatment decisions. Functionally, ARF6 enhances AML cell survival and proliferation while inhibiting apoptosis, highlighting its potential as a therapeutic target. Our outcomes offer new perspectives into the molecular pathways that govern AML etiology and development, paving the way for future research on ARF6‐targeted therapies in this aggressive malignancy.

## Author Contributions

Haitao Xu and Jian Ge conceptualized and supervised the research. Haitao Xu conducted most of the essays and authored the article. Dangui Chen and Jia Lu were accountable for the statistical analysis. Lihong Wang and Long Zhong revised the manuscript. All authors evaluated and accepted the final version of the manuscript.

## Ethics Statement

The patients were apprised of the collection and utilization of samples. The samples were gathered and employed aligning with the authorization granted by the Medical Ethics Committee of Anqing Municipal Hospital (ethics approval no. 202160).

## Conflicts of Interest

The authors declare no conflicts of interest.

## Supporting information


**Table S1.** Sequences used in this research.

**Table S2.** Primers used in plasmid construction.

## Data Availability

In the present study, publicly accessible datasets were subjected to analysis. The relevant data can be located at the hyperlinks provided below: https://portal.gdc.cancer.gov/; https://www.ncbi.nlm.nih.gov/geo/.

## References

[1] S. Shimony , M. Stahl , and R. M. Stone , “Acute Myeloid Leukemia: 2023 Update on Diagnosis, Risk‐Stratification, and Management,” American Journal of Hematology 98 (2023): 502–526. 36594187 10.1002/ajh.26822

[2] C. D. DiNardo , H. P. Erba , S. D. Freeman , and A. H. Wei , “Acute Myeloid Leukaemia,” Lancet 401 (2023): 2073–2086. 37068505 10.1016/S0140-6736(23)00108-3

[3] J. P. Bewersdorf and O. Abdel‐Wahab , “Translating Recent Advances in the Pathogenesis of Acute Myeloid Leukemia to the Clinic,” Genes & Development 36 (2022): 259–277. 35318270 10.1101/gad.349368.122 PMC8973851

[4] H. M. Kantarjian , T. M. Kadia , C. D. DiNardo , M. A. Welch , and F. Ravandi , “Acute Myeloid Leukemia: Treatment and Research Outlook for 2021 and the MD Anderson Approach,” Cancer 127 (2021): 1186–1207. 33734442 10.1002/cncr.33477 PMC12084862

[5] E. Peroni , M. L. Randi , A. Rosato , and S. Cagnin , “Acute Myeloid Leukemia: From NGS, Through scRNA‐Seq, to CAR‐T. Dissect Cancer Heterogeneity and Tailor the Treatment,” Journal of Experimental & Clinical Cancer Research 42, no. 1 (2023): 259. 37803464 10.1186/s13046-023-02841-8 PMC10557350

[6] N. Jahn , E. Jahn , M. Saadati , et al., “Genomic Landscape of Patients With FLT3‐Mutated Acute Myeloid Leukemia (AML) Treated Within the CALGB 10603/RATIFY Trial,” Leukemia 36 (2022): 2218–2227. 35922444 10.1038/s41375-022-01650-w PMC9417991

[7] Q. Zhang , B. Riley‐Gillis , L. Han , et al., “Activation of RAS/MAPK Pathway Confers MCL‐1 Mediated Acquired Resistance to BCL‐2 Inhibitor Venetoclax in Acute Myeloid Leukemia,” Signal Transduction and Targeted Therapy 7, no. 1 (2022): 51. 35185150 10.1038/s41392-021-00870-3 PMC8858957

[8] M. Tashakori , T. Kadia , S. Loghavi , et al., “TP53 Copy Number and Protein Expression Inform Mutation Status Across Risk Categories in Acute Myeloid Leukemia,” Blood 140 (2022): 58–72. 35390143 10.1182/blood.2021013983 PMC9346958

[9] K. Zaoui , C. V. Rajadurai , S. Duhamel , and M. Park , “Arf6 Regulates RhoB Subcellular Localization to Control Cancer Cell Invasion,” Journal of Cell Biology 218 (2019): 3812–3826. 31591185 10.1083/jcb.201806111 PMC6829653

[10] S. Kushwaha , B. Mallik , A. Bisht , et al., “Dasap Regulates Cellular Protrusions via an Arf6‐Dependent Actin Regulatory Pathway in S2R+ Cells,” FEBS Letters 598 (2024): 1491–1505. 38862211 10.1002/1873-3468.14954

[11] H. Sabe , “KRAS, MYC, and ARF6: Inseparable Relationships Cooperatively Promote Cancer Malignancy and Immune Evasion,” Cell Communication and Signaling: CCS 21 (2023): 106. 37158894 10.1186/s12964-023-01130-3 PMC10165578

[12] D. Sun , Y. Guo , P. Tang , H. Li , and L. Chen , “Arf6 as a Therapeutic Target: Structure, Mechanism, and Inhibitors,” Acta Pharmaceutica Sinica B 13 (2023): 4089–4104. 37799386 10.1016/j.apsb.2023.06.008 PMC10547916

[13] S. M. Luttgenau , C. Emming , T. Wagner , et al., “Pals1 Prevents Rac1‐Dependent Colorectal Cancer Cell Metastasis by Inhibiting Arf6,” Molecular Cancer 20 (2021): 74. 33941200 10.1186/s12943-021-01354-2 PMC8094600

[14] W. Wiese , J. Barczuk , O. Racinska , et al., “PI3K/Akt/mTOR Signaling Pathway in Blood Malignancies‐New Therapeutic Possibilities,” Cancers (Basel) 15, no. 21 (2023): 5297. 37958470 10.3390/cancers15215297 PMC10648005

[15] W. Y. Su , L. Y. Tian , L. P. Guo , L. Q. Huang , and W. Y. Gao , “PI3K Signaling‐Regulated Metabolic Reprogramming: From Mechanism to Application,” Biochimica Et Biophysica Acta. Reviews on Cancer 1878 (2023): 188952. 37499988 10.1016/j.bbcan.2023.188952

[16] A. Glaviano , A. S. C. Foo , H. Y. Lam , et al., “PI3K/AKT/mTOR Signaling Transduction Pathway and Targeted Therapies in Cancer,” Molecular Cancer 22 (2023): 138. 37596643 10.1186/s12943-023-01827-6 PMC10436543

[17] I. Nepstad , K. J. Hatfield , I. S. Gronningsaeter , and H. Reikvam , “The PI3K‐Akt‐mTOR Signaling Pathway in Human Acute Myeloid Leukemia (AML) Cells,” International Journal of Molecular Sciences 21 (2020): 2907. 32326335 10.3390/ijms21082907 PMC7215987

[18] K. Wang , Z. Ou , G. Deng , et al., “The Translational Landscape Revealed the Sequential Treatment Containing ATRA Plus PI3K/AKT Inhibitors as an Efficient Strategy for AML Therapy,” Pharmaceutics 14, no. 11 (2022): 2329. 36365147 10.3390/pharmaceutics14112329 PMC9696193

[19] K. Zhang , R. Huang , M. Ji , et al., “Rational Design and Optimization of Novel 4‐Methyl Quinazoline Derivatives as PI3K/HDAC Dual Inhibitors With Benzamide as Zinc Binding Moiety for the Treatment of Acute Myeloid Leukemia,” European Journal of Medicinal Chemistry 264 (2024): 116015. 38048697 10.1016/j.ejmech.2023.116015

[20] X. Huang , S. Lai , F. Qu , et al., “CCL18 Promotes Breast Cancer Progression by Exosomal miR‐760 Activation of ARF6/Src/PI3K/Akt Pathway,” Molecular Therapy–Oncolytics 25 (2022): 1–15. 35399607 10.1016/j.omto.2022.03.004 PMC8971730

[21] E. Fiola‐Masson , J. Artigalas , S. Campbell , and A. Claing , “Activation of the GTPase ARF6 Regulates Invasion of Human Vascular Smooth Muscle Cells by Stimulating MMP14 Activity,” Scientific Reports 12 (2022): 9532. 35680971 10.1038/s41598-022-13574-7 PMC9184495

[22] A. Colaprico , T. C. Silva , C. Olsen , et al., “TCGAbiolinks: An R/Bioconductor Package for Integrative Analysis of TCGA Data,” Nucleic Acids Research 44 (2016): e71. 26704973 10.1093/nar/gkv1507 PMC4856967

[23] X. Tu , H. Huang , S. Xu , C. Li , and S. Luo , “Single‐Cell Transcriptomics Reveals Immune Infiltrate in Sepsis,” Frontiers in Pharmacology 14 (2023): 1133145. 37113759 10.3389/fphar.2023.1133145 PMC10126435

[24] M. Xu , H. Zhou , P. Hu , et al., “Identification and Validation of Immune and Oxidative Stress‐Related Diagnostic Markers for Diabetic Nephropathy by WGCNA and Machine Learning,” Frontiers in Immunology 14 (2023): 1084531. 36911691 10.3389/fimmu.2023.1084531 PMC9992203

[25] J. Jiang , J. Feng , X. Song , et al., “Hsa_circ_0015278 Regulates FLT3‐ITD AML Progression via Ferroptosis‐Related Genes,” Cancers (Basel) 15, no. 1 (2022): 71. 36612069 10.3390/cancers15010071 PMC9817690

[26] P. Carella , “Close Encounters of the ARF Kind: Proximity‐Based ARF1 GTPase Activity Regulates Vesicle Trafficking,” Plant Cell 32 (2020): 2453–2454. 32554624 10.1105/tpc.20.00469 PMC7401020

[27] J. Fernandez‐Chamorro , R. Francisco‐Velilla , J. Ramajo , and E. Martinez‐Salas , “Rab1b and ARF5 Are Novel RNA‐Binding Proteins Involved in FMDV IRES‐Driven RNA Localization,” Life Science Alliance 2, no. 1 (2019): e201800131. 30655362 10.26508/lsa.201800131 PMC6337736

[28] Z. Ye , Q. Hu , Q. Zhuo , et al., “Abrogation of ARF6 Promotes RSL3‐Induced Ferroptosis and Mitigates Gemcitabine Resistance in Pancreatic Cancer Cells,” American Journal of Cancer Research 10 (2020): 1182–1193. 32368394 PMC7191101

[29] Y. Hu , Y. Huang , X. Xie , L. Li , Y. Zhang , and X. Zhang , “ARF6 Promotes Hepatocellular Carcinoma Proliferation Through Activating STAT3 Signaling,” Cancer Cell International 23 (2023): 205. 37716993 10.1186/s12935-023-03053-y PMC10505330

[30] S. Hashimoto , S. Mikami , H. Sugino , et al., “Lysophosphatidic Acid Activates Arf6 to Promote the Mesenchymal Malignancy of Renal Cancer,” Nature Communications 7 (2016): 10656. 10.1038/ncomms10656 PMC4748122 26854204

[31] C. Liang , Y. Qin , B. Zhang , et al., “ARF6, Induced by Mutant Kras, Promotes Proliferation and Warburg Effect in Pancreatic Cancer,” Cancer Letters 388 (2017): 303–311. 28025100 10.1016/j.canlet.2016.12.014

[32] B. Xiao , Y. Zhang , Z. Lu , et al., “A Positive Feedback Loop of ARF6 Activates ERK1/2 Signaling Pathway via DUSP6 Silencing to Promote Pancreatic Cancer Progression,” Acta Biochimica et Biophysica Sinica Shanghai 54 (2022): 1431–1440. 10.3724/abbs.2022111 PMC9827993 36017891

[33] G. R. Fajardo‐Orduna , E. Ledesma‐Martinez , I. Aguiniga‐Sanchez , M. L. Mora‐Garcia , B. Weiss‐Steider , and E. Santiago‐Osorio , “Inhibitors of Chemoresistance Pathways in Combination With Ara‐C to Overcome Multidrug Resistance in AML. A Mini Review,” International Journal of Molecular Sciences 22, no. 9 (2021): 4955. 34066940 10.3390/ijms22094955 PMC8124548

[34] Y. Ye , M. Labopin , S. Gerard , et al., “Lower Relapse Incidence With Haploidentical Versus Matched Sibling or Unrelated Donor Hematopoietic Cell Transplantation for Core‐Binding Factor AML Patients in CR2: A Study From the Global Committee and the Acute Leukemia Working Party of the European Society for Blood and Marrow Transplantation,” American Journal of Hematology 99 (2024): 1290–1299. 38654658 10.1002/ajh.27342

[35] L. Liu , L. Yang , X. Liu , et al., “SEMA4D/PlexinB1 Promotes AML Progression via Activation of PI3K/Akt Signaling,” Journal of Translational Medicine 20 (2022): 304. 35794581 10.1186/s12967-022-03500-w PMC9258142

[36] H. Gu , C. Chen , Z. S. Hou , et al., “PI3Kgamma Maintains the Self‐Renewal of Acute Myeloid Leukemia Stem Cells by Regulating the Pentose Phosphate Pathway,” Blood 143 (2024): 1965–1979. 38271660 10.1182/blood.2023022202 PMC11103183

[37] S. Hashimoto , S. Furukawa , A. Hashimoto , et al., “ARF6 and AMAP1 Are Major Targets of KRAS and TP53 Mutations to Promote Invasion, PD‐L1 Dynamics, and Immune Evasion of Pancreatic Cancer,” Proceedings of the National Academy of Sciences of the United States of America 116 (2019): 17450–17459. 31399545 10.1073/pnas.1901765116 PMC6717289

[38] A. Hashimoto and S. Hashimoto , “ADP‐Ribosylation Factor 6 Pathway Acts as a Key Executor of Mesenchymal Tumor Plasticity,” International Journal of Molecular Sciences 24 (2023): 14934. 37834383 10.3390/ijms241914934 PMC10573442

