# Supplementary material for: ARF6 Promotes AML Progression via Activation of PI3K/AKT/mTOR Signaling
Source: Cancer Med. 2025 Apr 24;14(9):e70872. doi: 10.1002/cam4.70872 (PMC12021670; doi:10.1002/cam4.70872)
Supplement: Supplementary file 1 — Table S1. Sequences used in this research. [file CAM4-14-e70872-s001.docx]

Supplementary Table 1. Sequences used in this research

| Gene | Sequences | |
| --- | --- | --- |
|  | sense（5'-3'） | antisense（5'-3'） |
| ARF6-Homo-803 | GUCUCAUCUUCGUAGUGGATT | UCCACUACGAAGAUGAGACTT |
| ARF6-Homo-895 | GACGCCAUAAUCCUCAUCUTT | AGAUGAGGAUUAUGGCGUCTT |
| ARF6-Homo-1051 | CUCACAUGGUUAACCUCUATT | UAGAGGUUAACCAUGUGAGTT |
| ARF6-Homo-584 | GGAACAAGGAAAUGCGGAUTT | AUCCGCAUUUCCUUGUUCCTT |
