# Supplementary material for: ARF6 Promotes AML Progression via Activation of PI3K/AKT/mTOR Signaling
Source: Cancer Med. 2025 Apr 24;14(9):e70872. doi: 10.1002/cam4.70872 (PMC12021670; doi:10.1002/cam4.70872)
Supplement: Supplementary file 2 — Table S2. Primers used in plasmid construction. [file CAM4-14-e70872-s002.docx]

Supplementary Table S2 Primers used in plasmid construction

| Homo ARF6-F | CTAGAGGATCTATTTCCGGTGAATTCATGGGGAAGGTGCTATCC |
| --- | --- |
| Homo ARF6-R | CGGGATCCGCGGCCGCTCTAGATTAAGATTTGTAGTTAGAGGTTAACC |
